# Regulators of Global Genome Repair Do Not Respond to DNA Damaging Therapy but Correlate with Survival in Melanoma

**DOI:** 10.1371/journal.pone.0070424

**Published:** 2013-08-05

**Authors:** Nikola A. Bowden, Katie A. Ashton, Ricardo E. Vilain, Kelly A. Avery-Kiejda, Ryan J. Davey, Heather C. Murray, Timothy Budden, Stephen G. Braye, Xu Dong Zhang, Peter Hersey, Rodney J. Scott

**Affiliations:** 1 School of Biomedical Sciences & Pharmacy, Faculty of Health, University of Newcastle, Australia and Hunter Medical Research Institute, Newcastle, NSW, Australia; 2 Hunter Area Pathology Service, John Hunter Hospital, Newcastle, NSW, Australia; 3 Kolling Institute, University of Sydney, NSW, Australia; Univesity of Texas Southwestern Medical Center at Dallas, United States of America

## Abstract

Nucleotide excision repair (NER) orchestrates the repair of helix distorting DNA damage, induced by both ultraviolet radiation (UVR) and cisplatin. There is evidence that the global genome repair (GGR) arm of NER is dysfunctional in melanoma and it is known to have limited induction in melanoma cell lines after cisplatin treatment. The aims of this study were to examine mRNA transcript levels of regulators of GGR and to investigate the downstream effect on global transcript expression in melanoma cell lines after cisplatin treatment and in melanoma tumours. The GGR regulators, *BRCA1* and *PCNA*, were induced in melanocytes after cisplatin, but not in melanoma cell lines. Transcripts associated with *BRCA1*, *BRCA2*, *ATM* and *CHEK2* showed altered expression in melanoma cell lines after cisplatin treatment. In melanoma tumour tissue *BRCA1* transcript expression correlated with poor survival and *XPB* expression correlated with solar elastosis levels. Taken together, these findings provide evidence of the mechanisms underlying NER deficiency in melanoma.

## Introduction

Nucleotide excision repair (NER) is primarily associated with the repair of the ultraviolet light radiation (UVR) induced lesions, cyclobutane pyrimidine dimers (CPD) and pyrimidine (6-4) pyrimidone photoproducts (6-4PP) [1]. Cisplatin is a common DNA-damaging agent that is used in the treatment of many types of malignancy [2]. Cisplatin binds to DNA forming similar helix distorting intra- and inter-strand cross-links [3], [4] which must be removed prior to either transcription or DNA replication. Accumulation of cisplatin-induced DNA damage results in cellular death. The removal and repair of large helix distorting DNA damage induced by cisplatin is orchestrated by NER [5]. The versatility of NER suggests that this mechanism may play a pivotal role in resistance to treatment and development of cancer.

The NER pathway consists of 2 DNA damage detection arms: Global genome repair (GGR) and transcription coupled repair (TCR). GGR operates across the entire genome and is a crucial step in the initial recognition of DNA damage [6]. GGR scans for damage in the non-coding regions of the genome, silent genes, and the non-transcribed strand of active genes [7], whereas TCR detects DNA damage in the transcribed strand of active genes using RNA polymerase II (RNAPII) as a lesion sensor [7]. Once the DNA damage is detected by GGR or TCR, the remaining members of the NER process are recruited. Briefly, this process involves unwinding of the DNA helix around the lesion by the helicases XPB and XPD, incision of the DNA upstream and downstream of the lesion by the endonucleases XPF/ERCC1 and XPG and DNA resynthesis and ligation by DNA polymerases δ and ε and DNA ligase I [8].

Even though NER is a vital component required for the maintenance of genomic integrity, studies investigating the role of NER in melanoma are limited. Current evidence suggests cells recognise cisplatin induced DNA damage but rather than repairing the lesions, NER triggers apoptosis [5]. If NER remained intact in melanoma cells, treatment with cisplatin should be highly effective but this is not the case. Therefore, it is possible that a reduced level of NER in melanoma may result in an accumulation of DNA damage rather than signalling apoptosis, which would be observed as a limited or absence of response to cisplatin treatment. The intra and inter-strand cross-links caused by cisplatin are recognised by the GGR component XPC then the DDB1/DDB2 complex is recruited to bind specifically to the large helix distorting DNA adducts [6]. We recently reported only a limited induction of *XPC*, *DDB1* and *DDB2* GGR transcripts in melanoma cell lines 24 hours after cisplatin treatment suggesting a breakdown in the normal NER response to DNA damage [9]. Other recent studies have sequenced the whole genome of a metastatic melanoma cell line [10] and 25 metastatic melanomas [11] to catalogue somatic mutations characteristic of melanoma. Both studies revealed the most frequent type of somatic mutation were C>T or CC>TT transitions at adjacent pyrimidines, indicative of residual UV DNA damage. Further, genome-wide investigation found a higher prevalence of the UVR mutational signature in lowly transcribed genes, suggesting that reduced activity of the GGR component of the NER pathway is predominantly responsible for the accumulation of the UVR mutational signature in melanoma. Despite this growing body of evidence, a recent study reported no difference in overall NER capacity between melanocytes and melanoma cell lines after UV-irradiation [12]. This seemingly contradictory finding may be due to melanocytes having a lower than normal NER capacity [13], which may indeed be the reason they are susceptible to malignant transformation after UV-irradiation. Irrespective of overall NER capacity being similar in melanocytes and melanoma cells, there is evidence that GGR is impaired in melanoma cells.

Despite this growing evidence, the underlying mechanisms have not been extensively investigated. Similarly, the downstream effects on transcription of reduced GGR capacity remain unclear. The present study examined gene transcripts involved in the regulation of GGR, after cisplatin treatment and investigated the downstream effects of a reduced GGR response in melanoma cell lines. The results of the cell culture analysis were correlated with events occurring in melanoma tissue. The results of this study shed light on the mechanisms by which GGR fails to be induced in response to cisplatin treatment in melanoma and we have also identified the possibility that GGR regulators may have potential as biomarkers of melanoma.

## Methods

### Ethics Statement

The use of diagnostic FFPE melanoma tissue in this study was approved by the Hunter New England Area Health Service Human Ethics Committee approval number 08/08/20/5.17. Waiver of written consent was obtained for the use of diagnostic fixed tissue (FFPE) blocks in this study by the Hunter New England Area Health Ethics Committee, therefore written informed consent is not available.

### Cell Lines and Cisplatin Treatment

One melanocyte, three primary melanoma (MM200, IgR3, Me4405) and two metastatic melanoma (Mel-RM and Sk-mel-28) cell lines were used for this study. The derivation of MM200, IgR3, Me4405, Mel-RM and Sk-Mel-28 melanoma cell lines has been described previously [14]–[17]. Sk-mel-28 has mutant *p53* and Me4405 was null for *p53* [14]. Melanocytes were purchased from Cascade Biologics at the commencement of this study. Cell line authentication was described previously [9], each cell line had a distinct individual set of markers present. All cell lines were routinely tested for mycoplasma every 3 months and were found to be free of contamination.

All of the melanoma cell lines were cultured in DMEM (5% FCS) and the melanocytes were cultured in Medium 254 (Cascade Biologics). All cell lines were maintained in exponential growth at 37°C and 5% CO_2_. Cells were treated with 10 µg/mL cisplatin (Pharmacia Upjohn) as previously described [14] and harvested before treatment and 6 and 24 hours after treatment for gene expression analysis. Total RNA was extracted and quantified as described previously [9]. Briefly, total RNA was extracted from all cell lines at all time points in duplicate using the SV Total RNA Isolation System (Promega).

### Melanoma Tumours

RNA was extracted from 196 formalin fixed paraffin embedded (FFPE) melanoma samples collected for diagnostic purposes at the Hunter Area Pathology Service, NSW, Australia between 2004 and 2008. The Hunter New England Area Health Service Human Ethics Committee approved the study. Clinical parameters of the tumours are outlined in Table 1.

**Table 1:** Clinical Characteristics of melanoma tumours.

Characteristics	Total Patient No. (%)
**Total**	157 (100)
**Sex**	
Female	48 (30.6)
Male	109 (69.4)
**Age at 1st Diagnosis**	
Mean (range)	65.8 (23.3–94.5)
Unknown	15 (9.6)
**Solar Elastosis**	
None	8 (5.1)
Mild	25 (15.9)
Moderate	17 (10.8)
Severe	42 (26.8)
Unknown	65 (41.4)
**Survival (weeks)**	
Mean (range)	206.6 (3.1–1418)
Alive	17 (10.8)
**Breslow Thickness**	
Mean (range)	5.3 (0.4–33)
No. Unknown	62 (39.5)
**Weeks Local to Distal Metastasis**	
Mean (range)	121 (5.6–343.4)
No. Unknown	66 (42)
**Mutation Status**	
BRAF	40 (25.5)
NRAS	33 (21.0)
Wildtype	84 (53.5)

### Illumina WGGEX arrays

Duplicate total RNA from all cell lines at all time points, was amplified and biotinylated using the Illumina TotalPrep kit (Ambion, USA). The resultant biotinylated cRNA was hybridised to Whole Genome Gene Expression Human Ref8 V3 BeadChips (Illumina, USA) containing approximately 24,000 transcripts. The BeadChips were scanned using a Bead Array Reader (Illumina USA).

The transcript expression results were cubic spline normalised using BeadStudio 2.0 software (Illumina, USA), and the remaining analyses was performed using GeneSpring GX 11.0. To account for bias or skewing of expression results all the gene expression profiles and each individual gene were normalized to the median resulting in two way normalisation. For visualisation of the results the data was log transformed. Raw and normalised data is available in the GEO repository (accession number GSE47980).

### Network diagram

GeneSpring GX 11.0 was used to build network diagrams of regulators and targets of the GGR transcripts. The relations used to build the networks are derived from published literature abstracts using a proprietary Natural Language Processing (NLP) algorithm and additional interactions from experimental data, available in public repositories (eg:IntAct).

Relation score was used as a measure of confidence in the relationship identified by the NLP algorithm. All relations derived from curated databases were given a score of 10 (highest score). NLP derived relationships were scored on a scale of 1–9, the best being 9 and the weakest being 1. The score is calculated based on the number of references, grade of the reference (known as RefScore, scale 1–9) and the syntax of the sentences. Any relation supported by at least one reference of RefScore 9 or having 3 or more references supporting it, is graded as 9. All relationships in the GGR network diagram generated for this study had a relation score >9 and were limited to the following interactions: binding, expression and regulation.

### Real-time PCR

RNA was reverse-transcribed and relative expression (RE) was measured as described previously [9]. Briefly, 500 ng of duplicate total RNA for each cell line at all timepoints was reverse-transcribed using the High Capacity Reverse Transcription kit (Applied Biosystems) and a 1∶20 dilution of the resultant cDNA was used in triplicate for each sample. For the melanoma tumours, 500 ng total RNA was reverse-transcribed and a 1∶20 dilution of cDNA was used in triplicate for each tumour. Relative expression was measured in triplicate and normalised to *β-actin* (ΔCt) using TaqMan gene expression assays (Applied Biosystems) and a 7500 real-time PCR system (applied Biosystems) for the following gene transcripts: *PCNA* and *BRCA1*. To ensure *β-actin* did not change between the cell lines/timepoints the ratio of *β-actin* to a second housekeeping gene *GAPDH* was measured. The average *β-actin/GAPDH* ratio was 1.02±0.04 across all the individual cell lines and treatment timepoints [9]. RE was calculated using 2^−ΔCt^ and unpaired, 2-tailed t-tests (p<0.05) were used to identify significantly altered expression as described previously [18]. Induction results are expressed as the normalised fold change induction of mRNA RE at 6 and 24 h compared to 0 h (which was set to 1). For the melanocytes, the results are the mean of triplicates of two independent experiments. For melanoma cell lines, the mean of triplicates of two independent experiments for each melanoma cell line was calculated, and the mean of all 5 cell lines was used for statistical analyses and for figures. For the melanoma tumours the mean of triplicates was used for analyses.

### Statistical analysis and gene set enrichment analysis (GSEA) using MSigDB

Significantly altered transcripts (P<0.05) in the melanocyte and melanoma cell lines at 24 hours compared to 0 hours were identified using Welch's T-test using Benjamin-Hochberg correction for multiple testing. Transcripts in common to both sets of altered transcripts were removed before further analyses to ensure specificity of analysis for the melanocytes versus the melanoma cell lines.

The Molecular Signatures Database (MSigDB) is a collection of annotated gene sets described in [19]. Overlap with lists of genes in the MSigDB was calculated for the lists of transcripts altered in the melanoma cell lines 24 hours after cisplatin treatment and the melanocytes 24 hours after cisplatin treatment compared to 0 hours. A gene set/list was considered as having highly significant overlap and included in further analyses if p<0.0001, p values for the MSigdB analysis were calculated using the hypergeometric test.

### Statistical analysis of PCNA, BRCA1 and XPB transcript expression in melanoma tumours

Correlation between *PCNA, BRCA1, XPB* transcript expression and the clinical parameters outlined in Table 1 was performed using both Spearman's Rho and Kendall's tau_b tests.

Kaplan-Meier survival analysis was used to plot cumulative survival versus weeks of survival (after first diagnosis) by high or low transcript expression as determined by above or below the median value respectively. Comparison of Kaplan-Meier survival curves was used to observe differences in survival based on high or low expression. The statistical analysis for survival was performed as described previously [20]. Briefly, Wilcoxon's test was used to determine the significance of the observed weeks of survival and the log-rank test and Tarone-Ware test were also used to test the homogeneity of the survival curves.

## Results

### GGR regulator and downstream target transcript expression

**Figure 1:**
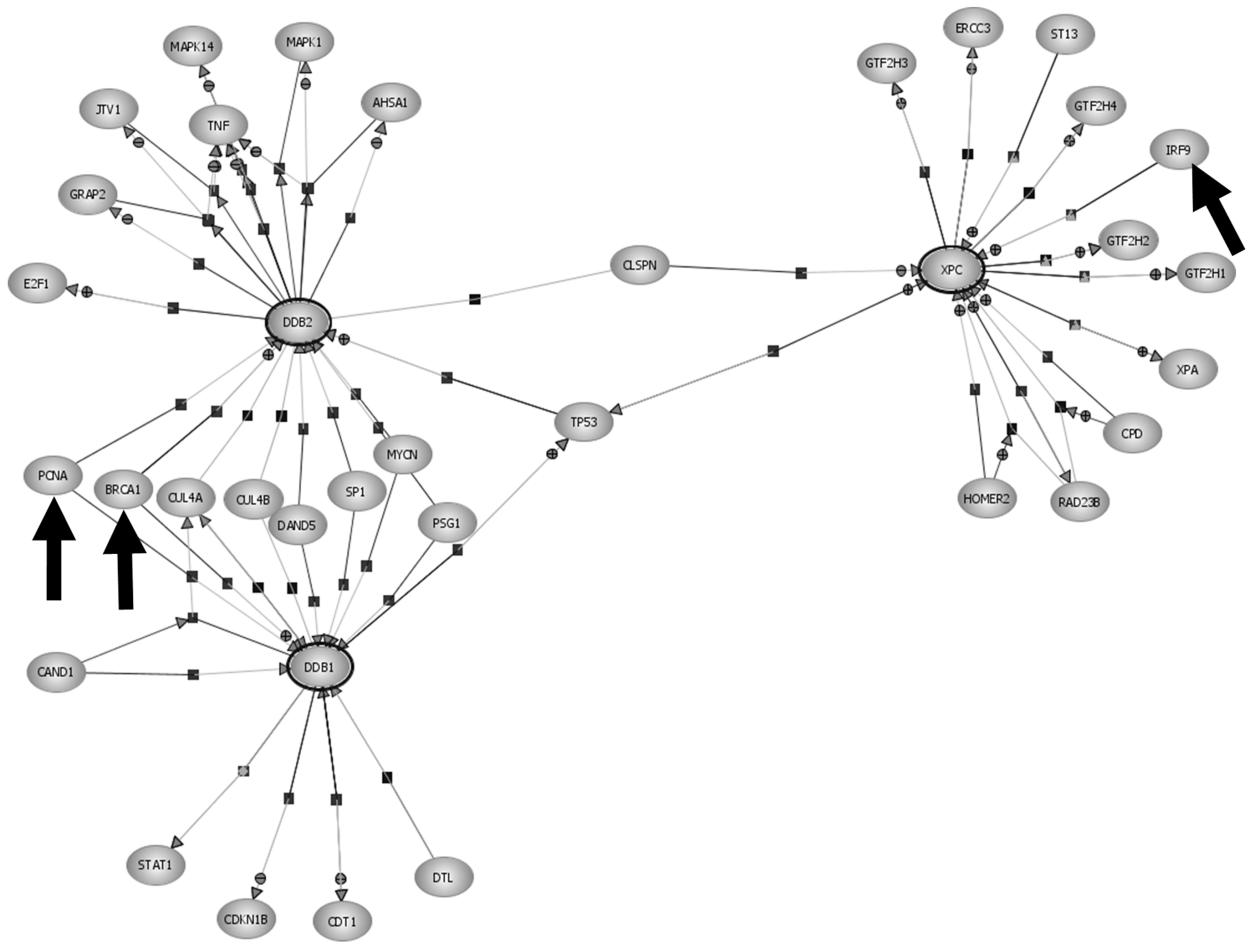
Network diagram of transcription regulators and targets of the GGR transcripts XPC, DDB1 and DDB2. Direction of small arrows represents direction of regulation. Regulators of XPC, DDB1 and DDB2 which had significantly higher expression 24 hrs after cisplatin treatment in the melanocytes but not the melanoma cell lines are indicated by large arrows.

A network of regulators of GGR and downstream targets of the three GGR transcripts was generated using a NLP algorithm (Figure 1). *p53* was the only transcript previously reported to have regulatory interaction at the protein level with all three GGR genes, *DDB1*, *DDB2* and *XPC* [21], [22]. We have previously reported the transcript expression levels of *p53* were not significantly different in the melanoma cell lines compared to the melanocyte cell line 24 hours after cisplatin treatment as assessed by real-time PCR analysis [9].

**Figure 2:**
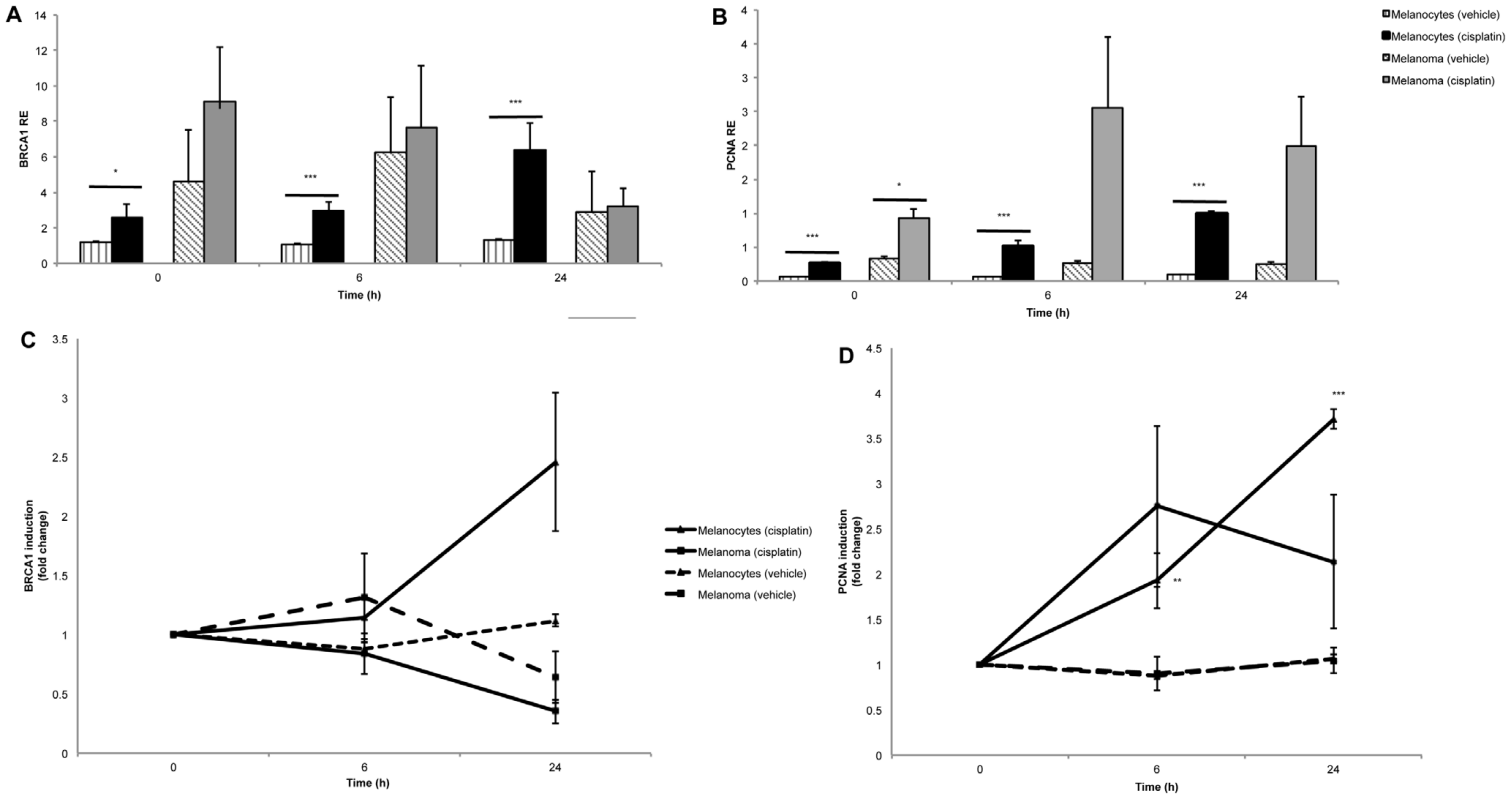
Relative expression (RE) and induction of BRCA1 and PCNA after cisplatin treatment in melanocytes and melanoma. A) RE of BRCA1 and PCNA at 0, 6, 24 h after cisplatin treatment in melanoma and melanocyte cell lines. The RE of BRCA1 (p = NS) and PCNA (p = 0.0004) was higher at the basal level and 6 hours after cisplatin treatment in the melanoma cell lines, but BRCA1 was lower 24 hours after treatment (p = NS). The difference between the melanoma and melanocyte cell lines was not significant at any of the time points for BRCA1. Points are the mean of triplicates of two independent experiments, bars = SE. B) Effect of cisplatin treatment on the induction of BRCA1 and PCNA in melanoma and melanocyte cell lines at 6 and 24 h after cisplatin treatment. After 24 h induction of BRCA1 was significant in melanocytes, but decreased (p = NS) at 6 and 24 h in the melanoma cell lines. PCNA induction increased significantly 6 h (p = 0.009) and 24 h (p = 5×10^−10^) after treatment in melanocytes. Similarly, PCNA induction occurred at 6 h and 24 h after treatment in a portion of the melanoma cell lines, but the induction was considerably variable and not significant. Results are expressed as the normalised fold change induction of mRNA RE at 0 h (which was set to 1). Points are mean of triplicates of two independent experiments, bars = SE. *p<0.05.

Importantly, DNA repair transcripts *PCNA* and *BRCA1*, both of which have previously been reported to regulate *DDB1* and *DDB2* transcript expression [23]–[26], had significantly higher induction in melanocytes 24 hours after cisplatin treatment but not in the majority of the melanoma cell lines (Figure 2 and Figure S1). The primary melanoma cell line IgR3 was the only melanoma cell line to show increased *BRCA1* expression at 24 hours, but it was largely variable with a fold change of 3.17±1.29 (Figure S1). There was no significant difference in *PCNA* or *BRCA1* expression between primary and metastatic melanoma cell lines or in the presence/absence of a functional p53 transcript.

The downstream targets of *DDB1* and *DDB2*, *STAT1* and *MAPK14*, respectively, showed slightly increased expression (*STAT1* 1.4 fold increase, p = 0.03, *MAPK14* 1.4 fold increase p = 0.05) in the melanocytes after cisplatin treatment. A third downstream target of *DDB1* and *DDB2*, *CDT1* had 1.4 fold decrease in expression (p = 0.007) 24 h after cisplatin treatment in melanocytes. None of the GGR downstream target transcripts identified by the NLP had significantly altered expression after cisplatin treatment in the melanoma cell lines.

### Genome-wide transcript expression and gene set enrichment analysis

The consequences of cisplatin treatment in the melanocytes and melanoma cell lines respectively, was investigated at the whole transcriptome level using gene expression data for ∼24,000 transcripts. 3663 transcripts had significantly altered expression levels in the melanocyte cell line 24 hours after cisplatin treatment when compared to baseline expression levels (treatment = 0 hours). 3650 transcripts had significantly altered expression levels in the melanoma cell lines 24 hours after cisplatin treatment. 1084 of these transcripts were altered in both melanocyte and melanoma cell lines and are most likely to be transcripts generally involved in cisplatin response, therefore they were removed before further analyses. The remaining 2566 and 2579 transcripts altered specifically in melanoma and melanocytes respectively were used for gene set enrichment analysis (GSEA) using the molecular signatures database (MSigDB) [19]. The melanocyte and melanoma gene sets were each individually tested for overlap with gene sets in the MSigDB. The 50 genes sets that most significantly overlapped the melanocyte or melanoma gene sets (p<0.001) were studied further to investigate potential mechanisms involved in the differences in gene expression in response to cisplatin in melanoma and melanocyte cell lines. Gene sets with the highest relevance are reported in Tables 2 and 3. The gene sets overlapping with the transcripts altered in melanocytes 24 hours after cisplatin treatment were mostly related to normal cellular response to apoptosis inducing stimuli, e.g.: reovirus infection, CD40 stimulation, up-regulation of TP53 and importantly, UVB irradiation. There was a highly significant overlap of genes altered in response to cisplatin treatment in melanocytes and genes altered in response to UVB irradiation in epidermis (p = 2.07×10^−10^) and normal epidermal keratinocytes (NHEK cells) (p = 1.43×10^−7^). Interestingly, there was also significant overlap with genes up and down regulated in class 2 vs. class 1 uveal melanoma.

**Table 2:** MSigDB gene sets that significantly overlap the set of transcripts altered in melanocytes and melanoma cell lines 24 hours after cisplatin treatment.

Transcripts altered in melanocytes 24 hours after cisplatin treatment.				
Description	# Genes in Overlap (k)	# Genes in Gene Set (K)	p value	Ref.
Genes up-regulated in uveal melanoma: class 2 vs. class 1 tumors.	141	793	1.23×10^−11^	[36]
Genes down-regulated in HEK293 cells after infection with reovirus strain T3A	56	227	1.75×10^−10^	[37]
Genes down-regulated in epidermis after UVB	99	515	2.07×10^−10^	[38]
Genes down-regulated in uveal melanoma: class 2 vs. class 1 tumors.	93	532	9.18×10^−8^	[36]
Genes up-regulated in normal epidermal keratinocytes after UVB	93	537	1.43×10^−7^	[38]
Genes up-regulated in primary mammary epithelium upon expression of TP53	174	1191	2.77×10^−7^	[39]
Genes down-regulated in diffuse large B-cell lymphoma cell lines sensitive to CD40 stimulation	55	271	3.53×10^−7^	[40]

Transcripts altered in melanoma 24 hours after cisplatin treatment.				
Description	# Genes in Overlap (k)	# Genes in Gene Set (K)	p value	Ref.
Genes positively correlated (PCC > = 0.4) with BRCA1	318	1671	<0.001×10^−20^	[41]
Genes positively correlated (PCC > = 0.4) with CHEK2	159	782	<0.001×10^−20^	[41]
Down-regulated in fibroblasts expressing mutant forms of ERCC3 after UV	244	855	<0.001×10^−20^	[42]
Down-regulated in WS1 (fibroblast) in response to high dose UV-C.	72	245	<0.001×10^−20^	[43]
Down-regulated transcripts in ERCC3 mutant fibroblasts after UVC	130	420	<0.001×10^−20^	[42]
Genes down-regulated in normal keratinocytes by UVB	81	261	<0.001×10^−20^	[44]
Genes showing cell-cycle stage-specific expression	129	648	4.77×10^−15^	[45]
Genes positively correlated (PCC > = 0.4) with BRCA2	95	432	4.60×10^−14^	[41]
Genes positively correlated (PCC > = 0.4) with ATM	231	1461	1.28×10^−13^	[41]
Reactome: Genes involved in Cell Cycle, Mitotic	71	306	5.32×10^−12^	[46]
Genes increasingly down-regulated in normal keratinocytes after UVB	40	125	7.62×10^−12^	[44]
Up-regulated in uveal melanoma: class 2 vs. class 1 tumors.	138	793	2.11×10^−11^	[36]
Up-regulated in normal epidermal keratinocytes after UVB	103	537	2.89×10^−11^	[38]
Up-regulated in ERCC3 mutant fibroblasts after UV	69	319	2.92×10^−10^	[42]
Genes down-regulated in normal epidermal keratinocytes after UVB	91	486	1.48×10^−9^	[38]

**Table 3:** Significant correlation of BRCA1 transcript expression with clinical parameters.

Correlation	BRCA1
**Survival (weeks)**	
Kendall's tau_b	0.183, p = 0.027
Spearman's Rho	0.223, p = 0.027
**Solar Elastosis**	
Kendall's tau_b	−0.248, p = 0.032
Spearman's Rho	−0.264, p = 0.031

The gene sets overlapping with the transcripts altered in melanoma cell lines 24 hours after cisplatin treatment were highly correlated with DNA repair and DNA damage response genes that included *BRCA1*, *BRCA2*, *ATM* and *CHEK2*. As well as these DDR genes there was a highly significant (p<1×10^−20^) overlap of genes with altered expression after UV irradiation in *ERCC3 (XPB)* mutant cells. *XPB* is a helicase involved in the NER pathway and mutations in this gene result in very low NER capacity.

Finally, a highly significant (p<1×10^−20^) overlap of genes altered in response to UVC irradiation in fibroblasts and UVB irradiation of normal epidermal keratinocytes (NHEK cells) was observed for the melanocyte cell line. This was also reflected in gene expression changes seen in class 2 vs. class 1 uveal melanoma.

### PCNA, BRCA1 and XPB expression in melanoma tumours

*PCNA*, *BRCA1* and *XPB* displayed a lack of induction and highly correlated with altered expression in melanoma after cisplatin treatment by GSEA, therefore they were further investigated in 157 primary and metastatic melanoma tumours. The melanoma tumours were derived from fixed tissue blocks previously used for pathological diagnoses. The clinical characteristics of the melanomas are summarised in Table 1. The correlation analysis between transcript expression and clinical characteristics revealed no correlation between *PCNA* and any clinical parameters. *BRCA1* transcript expression correlated with disease specific survival and solar elastosis (Table 3). *XPB* showed a trend towards correlation to the level of solar elastosis compared to disease stage or survival.

**Figure 3:**
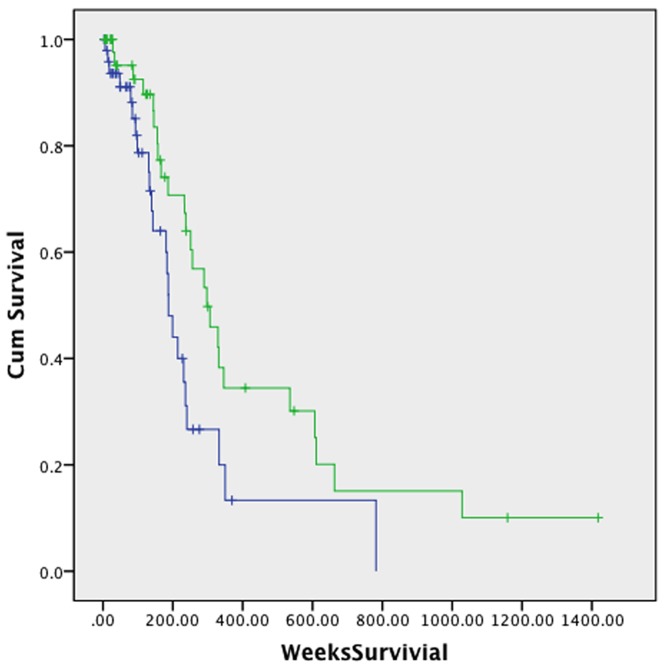
Kaplan-Meier Plot of Survival for BRCA1 transcript expression in melanoma tumours. Kaplan-Meier survival analysis was used to plot cumulative survival versus weeks of survival (after first diagnosis) by high or low *BRCA1* transcript expression as determined by above or below the median value respectively. Low *BRCA1* expression was significantly related to poor survival with an average of 260.4±47.9 weeks survival compared to 453.9±77.5 weeks for high *BRCA1* expression (p = 0.02).

Subsequently, expression of *PCNA*, *BRCA1* and *XPB* transcripts were used for Kaplan-Meier survival analyses. High versus low (determined by above or below the median) *XPB* or *PCNA* transcript expression did not appear to be correlated with survival, although *PCNA* did show a non-significant trend towards higher expression and poorer survival. Low expression of *BRCA1* however, was significantly related to poor survival. Although there was large variation in survival, low *BRCA1* expression was significantly related with an average of 260.4±47.9 weeks survival compared to 453.9±77.5 weeks for high *BRCA1* expression (p = 0.02) (Figure 3).

## Discussion

Despite growing evidence that GGR may play a role in cisplatin resistance, the underlying cause of this has not been investigated. Similarly, the downstream effects on transcription of reduced GGR and subsequent NER have also not been thoroughly examined. There is a strong correlation between reduced *XPC* mRNA and protein levels and increased resistance of cancer cells to cisplatin treatment [27]–[29]. More recently, it has been reported that in addition to its role in DNA damage recognition, *DDB2* is a key determinant in deciding the fate of a cell after DNA damage. Stoyanova and colleagues (2009) reported that wildtype mouse embryonic fibroblasts (MEFs) undergo apoptosis in response to both UVR and cisplatin but *DDB2* −/− MEFs show a much lower level of apoptotic response. This led to the discovery that in the absence of *DDB2*, cells undergo cell cycle arrest rather than apoptosis [30]. The transcriptional regulation of *XPC*, *DDB1* and *DDB2* in response to DNA-damaging agents such as cisplatin and UV-irradiation is yet to be fully investigated.

Using a NLP algorithm and whole transcriptome gene expression data, we identified *p53*, *BRCA1* and *PCNA* as transcriptional regulators of GGR. *p53* was induced in melanocytes after cisplatin treatment in our previous study [9] and *BRCA1* and *PCNA* were significantly induced in melanocytes after cisplatin in this study. *p53*, *BRCA1* and *PCNA* were not induced in the melanoma cell lines in response to cisplatin. It is known that p53 sustains higher basal levels of the p48 component of the DDB complex, and upregulates its expression in response to DNA damage [31]. It has also been reported that following UV irradiation p53 upregulates the XPC protein, as part of the GGR response [32]. From these observations it is clear that p53 can transactivate NER under normal circumstances, but may be aberrant in melanoma.

BRCA1 is known to regulate GGR in a p53-independent manner via GADD45 and plays a compensatory role for inducing GGR and subsequent apoptosis in the absence of p53 [25]. The limited induction of *p53* in melanoma cell lines after cisplatin treatment [9] and the limited induction of *BRCA1* reported herein confirms that the two compensatory regulators primarily responsible for GGR induction are both deficient in melanoma. PCNA is a DNA clamp and co-factor for DNA polymerase δ, which together are involved in translesion synthesis in the NER pathway. PCNA is also known to physically interact with several complexes including the CUL4/DDB1/DDB2 complex which, in addition to recognising DNA damage in non-transcribed regions of the genome, regulates polyubiquitination of p53 and proteolysis of MDM2 and CDT1 after DNA damage [23]. *CDT1* was significantly reduced at the mRNA level 24 h after cisplatin treatment in the melanocytes, suggesting that induction of *PCNA* may limit transcription of *CDT1* in addition to increasing CDT1 proteolysis after DNA damage. Significant induction of *PCNA* in response to cisplatin treatment in melanocytes in this study further confirms the role of PCNA in DNA damage response, but it is not clear if the absence of *PCNA* induction in the melanoma cell lines is a consequence or cause of the absence of GGR induction. The limited induction of *PCNA* may be responsible for the limited GGR response to cisplatin-induced DNA damage in melanoma. The *p53, BRCA1, PCNA* and subsequent GGR induction in the melanocytes but not melanoma cell lines strongly supports the evidence that these transcripts control the GGR response to damage in a normal cellular system but are impaired in melanoma. Further studies in additional melanocyte cell lines are required to conclusively confirm this finding.

The generation of the NLP network of regulators and transcripts provided the basis for further investigation into the regulation of GGR. We undertook global transcript analysis of the response to cisplatin treatment that did not rely on *a priori* knowledge, rather it was completely dependent on statistical analysis of transcript expression. Transcripts with altered expression in the melanocytes were very similar to the normal cellular response to apoptosis inducing stimuli such as reovirus, CD40 stimulation, up-regulation of p53 and UVB irradiation. The similarity to UVB irradiation of normal epidermal keratinocytes and epidermis is not surprising as both UVB and cisplatin induce helix-distorting DNA damage that is repaired by NER.

The gene sets with significant overlap of transcripts altered in the melanoma cell lines 24 h after cisplatin treatment was quite different to the melanocytes. Although there was overlap with the response of fibroblasts and keratinocytes to UVR there was a greater overlap with transcripts that correlate highly with expression of the DNA double strand break (DSB) repair genes *BRCA1* and *BRCA2* and the DNA damage response genes *ATM* and *CHEK2*. The exact cause of this overlap in transcripts is unknown but given that there is a certain level of redundancy between DNA repair processes, it is feasible that double strand break (DSB) repair may be compensating for the NER deficiency in the melanoma cell lines. Given that we have identified limited induction of *BRCA1* in the melanoma cell lines in response to cisplatin in this study, the result was not unexpected and may be indicative of other *BRCA1*-associated DNA repair transcripts undergoing normal response to DNA-damage inducing stimuli. Further support for the role of BRCA1 in DDR deficiency was the low *BRCA1* expression in melanomas correlating with poor survival as shown by Kaplan-Meier analysis. Although this finding is irrespective of treatment, it is tantalising and requires further investigation given recent reports of BRCA1 being overexpressed in melanoma non-responders to chemotherapy [33] and patients with melanoma relapse [34].

The very significant overlap of transcripts differentially regulated in ERCC3 (XPB) deficient cells after UVR was the most striking of the gene sets overlapping with the transcripts altered in response to cisplatin in the melanoma cell lines. The previously reported absence of *XPB* induction [9] and the highly significant overlap with transcripts altered in XPB deficient fibroblasts after UVR is highly suggestive of melanoma cells having a very limited NER capacity of somewhere between 3% and 7% of normal as reported in XPB deficient fibroblasts [35]. Given that one of the key clinical features of individuals with mutations in the *XPB* gene is UVR sensitivity and an increase in UV-induced melanomas [35], the role of this gene in melanomagenesis and cisplatin resistance in the general population requires further investigation.

### Conclusions

In this study we have confirmed that the GGR regulators, *BRCA1* and *PCNA*, are induced in the normal cellular response to cisplatin induced DNA damage, but there is complete absence of induction of these regulators in melanoma cell lines. A highly significant overlap of transcript expression in melanoma cell lines after cisplatin treatment with transcripts involved in DNA repair and DNA damage response genes *BRCA1*, *BRCA2*, *ATM* and *CHEK2* was observed, which may be compensating for diminished NER capacity. We also identified a significant overlap of transcript expression with XPB deficient cells after UVR. Finally, we investigated correlation between *PCNA*, *BRCA1* and *XPB* transcript expression and clinical parameters and found that low *BRCA1* expression is significantly associated with poor survival. Taken together these findings provide support for the role of *BRCA1* and to a lesser extent *PCNA*, in regulating NER in melanomagenesis and resistance to cisplatin treatment.

## Supporting Information

Figure S1 **Induction of BRCA1 and PCNA after cisplatin treatment in melanocytes and melanoma cell lines.** Induction of BRCA1 and PCNA at 0, 6, 24 h after cisplatin treatment in individual melanoma and melanocyte cell lines. Points are the mean of triplicates of two independent experiments, bars = SE.
(TIFF)
